# A Study of Biofeedback Gait Training in Cerebral Stroke Patients in the Early Recovery Phase with Stance Phase as Target Parameter

**DOI:** 10.3390/s21217217

**Published:** 2021-10-29

**Authors:** Dmitry V. Skvortsov, Sergey N. Kaurkin, Galina E. Ivanova

**Affiliations:** 1 Federal State Budgetary Institution «Federal Center of Brain Research and Neurotechnologies» of the Federal Medical Biological Agency, 117997 Moscow, Russia; kaurkins@bk.ru (S.N.K.); reabilivanova@mail.ru (G.E.I.); 2 Pirogov Russian National Research Medical University, 117997 Moscow, Russia; 3 Federal Research and Clinical Centre of Russia’s Federal Medical-Biological Agency (FNKC FMBA), 115682 Moscow, Russia

**Keywords:** biofeedback gait training, gait analysis, stroke, early recovery phase

## Abstract

Walking function disorders are typical for patients after cerebral stroke. Biofeedback technology (BFB) is currently considered effective and promising for training walking function, including in patients after cerebral stroke. Most studies recognize that BFB training is a promising tool for improving walking function; however, the data on the use of highly selective walking parameters for BFB training are very limited. The aim of our study was to investigate the feasibility of using BFB training targeting one of the basic parameters of gait symmetry—stance phase duration—in cerebral stroke patients in the early recovery period. The study included 20 hemiparetic patients in the early recovery period after the first hemispheric ischemic stroke. The control group included 20 healthy subjects. The BFB training and biomechanical analysis of walking (before and after all BFB sessions) were done using an inertial system. The mean number of BFB sessions was nine (from 8 to 11) during the three weeks in clinic. There was not a single negative response to BFB training among the study patients, either during the sessions or later. The spatiotemporal parameters of walking showed the whole syndrome complex of slow walking and typical asymmetry of temporal walking parameters, and did not change significantly as a result of the study therapy. The changes were more significant for the functioning of hip and knee joints. The contralateral hip amplitude returned to the normal range. For the knee joint, the amplitude of the first flexion increased and the value of the amplitude of hyperextension decreased in the middle of the stance phase. Concerning muscle function, the observed significant decrease in the function of m. Gastrocnemius and the hamstring muscles on the paretic side remained without change at the end of the treatment course. We obtained positive dynamics of the biomechanical parameters of walking in patients after the BFB training course. The feasibility and efficacy of their use for targeted correction need further research.

## 1. Introduction

Biofeedback technology (BFB) is currently considered effective and promising for training walking function [1,2,3], including in patients after cerebral stroke [3]. The technology is based on capturing a physiological parameter and presenting it to the patient in a perceivable form, so that the subject can understand its changes and respond appropriately. BFB can be used independently or as part of rehabilitation therapy. BFB can activate the body’s own resources, with the patient taking active part in the process of his or her recovery. Different types of BFB rely on different technical methods and senses. The most frequently used feedback channel is vision, followed by hearing alone or in combination with vision, whereas kinesthetic feedback is much more rare [1,4].

Walking function disorders are typical for patients after cerebral stroke. A cerebral stroke normally affects one hemisphere and causes a hemiplegic syndrome [5]. The gait of hemiplegic patients has very specific features [6,7]: reduced walking speed, increased double stance phase, and reduced amplitude of movement in the leg joints [8]. There are four main gait abnormalities associated with hemiparesis [9]: the drop-foot gait, the circumduction gait, the hip hiking gait, and the back knee gait.

BFB technology was not used for improving walking function until recently because of the technical complexity of capturing the basic gait parameters. The most often used parameters are walking speed, stride length, and cadence of walking [1]. It should be noted that these are the main resultant parameters of walking as the function of the body’s locomotion in space. However, walking speed will depend on other changes which determine the existing motor impairment: e.g., the presence of joint contractures, impaired muscle function, pain syndrome associated with certain movements or axial load on the limb, etc. In addition, the most frequently used parameter—walking speed—is not specific to a particular disease or condition, and the same is true for cadence. Walking speed can decrease as a result of a variety of pathological conditions. For all its importance, this parameter is not specific to the affected side. In contrast, the less often-used stride length may differ between the left and right sides. The third parameter—cadence—is also not specific to the affected side. However, not only do the above mentioned resultant parameters change significantly in patients with cerebral stroke but also their determinant parameters [8,9,10]: walking cycle duration, stance period, double and single support periods, and gait harmony (equal intervals of the start of the walking cycle (WC) on both sides).

In some cases, BFB training equipment can capture individual parameters: dynamic load on the limb [11,12] or the clearance between the foot and the ground [13]. Another commonly used parameter is the EMG activity of muscles (mostly of the lower-leg triceps). Spatiotemporal, kinematic, and kinetic parameters are also used [3]. Spatiotemporal parameters are most often used to train step length on the paretic side [3].

In recent years, owing to its important advantages, wearable IMU technology (systems using inertial measurement units) has been widely applied for capturing biomechanical gait parameters. A review of publications on the validation of IMU technology for capturing basic gait parameters in healthy adults [14] has shown high validity for some sets of parameters. Attempts are being made to automatically classify post-stroke walking abnormalities using IMUs and neural networks [15]. Some authors report using this technology for BFB therapy in patients with the sequelae of cerebral stroke and Parkinson’s disease [16]. However, according to a systematic review [16], IMU-based BFB systems showed good results only for balance training. There is still little information regarding other motor skills. Where walking function is concerned, the use of IMUs theoretically allows for a more selective choice of parameters for BFB training, but the available information is not sufficient to judge the feasibility and efficacy of such training.

The use of BFB to restore walking function in cerebral stroke patients had contradictory results, even with the same authors. In a study carried out by Druzbicki et al. [17], no significant effect of BFB training was noted. However, some effect was noted in their earlier study [18]. In the research of Gente et al. [12], training was based on the ground reaction force at the end of the stance phase, with both visual and auditory feedback. A training session lasted as little as six minutes. The obtained results demonstrated a significant increase in the developed effort. We found only one study [19] where stance phase was successfully used as the BFB parameter. The system used vibrotactile devices to provide the feedback. The authors noted that stance phase as the feedback parameter was more effective than healthy-to-paretic-side ratio. The authors used an experimental system created for that study.

The available literature does not offer a common standard approach to BFB training of walking in cerebral stroke patients. Technologies such as BFB typically provide a certain number of similar training sessions of a certain duration. There is a wide spread in the values of training parameters in the literature. In one review [3], the frequency of training sessions ranged from thrice a week to twice a day. The total number of sessions ranged from three to 18, with a significant number of single training sessions. Multi-session training typically included 10 to 20 sessions. The authors noted that this data spread could be due to the limited data in the reviewed literature.

Another study [17] included 15 training sessions. At the same time, there were reports indicating the efficacy of only one training session [12]. That session, however, was targeted at a condition-specific parameter—ground reaction force (dynamic load).

The duration of a training session also varied considerably. In the analytical review by Spencer et al. [3], session duration ranged from 11 to 30 min. In a study by Gente et al. [12], the training session lasted 30 min, and in another study by Begg et al. [13] only 10 min.

Thus, the relevant literature so far does not provide any objective criteria for conducting BFB training, including the number of training sessions, their duration, and the inter-session intervals.

The use of various types of BFB training to improve walking in patients with cerebral palsy also had contradictory results [17]. In that study, 30 patients with subacute cerebral stroke received treadmill walking training with or without the use of BFB based on kinetic parameters (15 patients per group). The results showed no differences in walking function between the two groups. Positive changes in walking biomechanics were noted in both groups. An earlier study by the same authors demonstrated improvement in gait symmetry and spatiotemporal parameters [18]. Other researchers [19] demonstrated a positive response to BFB training using a specific parameter (stance phase).

In general, most studies recognize that BFB training is a promising tool to improve walking function, especially in patients with cerebral stroke. At the same time, the data on the use of highly selective walking parameters for BFB training are very limited, which is obviously explained by the technological complexity of their use in such training. In addition, the main training parameters—session frequency, duration, exercise load, and performance criteria—are still not clear.

The aim of our study was to investigate the feasibility of using BFB training targeting one of the basic parameters of gait symmetry—stance phase duration—in cerebral stroke patients in the early recovery period.

Our hypothesis is that targeted training with the BFB walking on a selected parameter will allow for the changing of this parameter.

## 2. Materials and Methods

The study was performed during 2020–2021 at the Clinical Biomechanics Laboratory of the Federal Center of Brain Research and Neurotechnologies, FMBA of Russia.

The study included 20 hemiparetic patients in the early recovery period after a first hemispheric ischemic stroke.

The study population included 15 males and five female subjects at a mean age of 49.05 ± 12.44 years (range: 23–65 years), with a mean height of 176.35 ± 8.52 cm (range: 159–195 cm) and a mean weight of 79.50 ± 13.34 kg (49–102 kg); 113.05 ± 47.96 (28–179) days after cerebrovascular accident; nine subjects with right and 11 with left hemisphere damage.

Inclusion criteria: first-time hemiparetic ischemic stroke patients in the early recovery period; age up to 75 years; functional readiness for verticalization; adequate orthostatic response; able to stand upright for a minute; able to walk without mobility aids; fully conscious and alert enough to understand and follow instructions during research and training; no cognitive impairment that might prevent understanding the study staff instructions; absence of sensorimotor aphasia; lower limb muscle tone > 2 according to the modified Ashwoth spasticity scale; absence of decompensated somatic pathology, absence of ischemic changes in ECG; absence of heart failure (Killip Class II and higher); absence of diseases of the central and peripheral nervous systems, other than stroke, associated with neurological deficit (sequelae of injuries, tumors, polyneuropathy, etc.); absence of orthopedic pathology (joint deformities and contractures, pronounced pain syndrome, amputated limbs, etc.).

Exclusion criteria: inadequate cardiovascular response to training; fear of walking on the treadmill; patient’s refusal of therapy; negative changes in neurological and/or somatic status.

The functional capabilities of the patients were assessed using the Timed Up and Go Test (TUG) [20] and the Hauser Ambulance Index (HAI) [21] for walking, and the Berg Balance Scale (BB) [22] and the Standing Balance Test (SBT) for balance [23].

The healthy control group included 20 healthy subjects (10 females and 10 males, mean age 28.8 ± 3.66 years (23–35 years), mean height 176.8 ± 5.53 cm (168–188 cm), mean weight 76.25 ± 14.09 kg (55–100 kg), without a history of injuries or musculoskeletal diseases.

Basic walking parameters normally remain stable from adolescence to the age of 70 [24,25]. Therefore, the younger age of the healthy control group is acceptable.

### 2.1. Assessment of Walking Function

**Figure 1:**
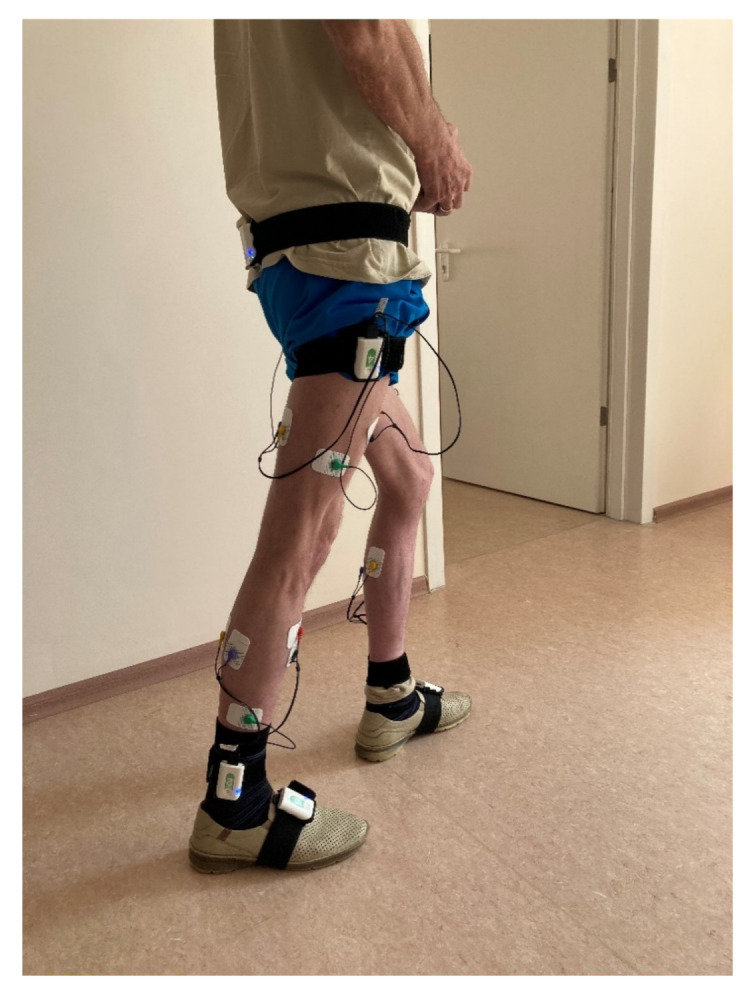
The patient during recording of the biomechanical parameters of walking. Inertial sensors were fixed on body segments (pelvis, hips, legs, feet). EMG electrodes were connected to the sensors on the thigh and shin, which were fixed on the corresponding muscles.

Biomechanical analysis of walking was performed using a Stadis system (Neurosoft, Ivanovo, Russia). Neurosens inertial sensors were attached to the subject’s sacrum, the outer middle third of the thigh, the outer ankle and the foot instep, on both sides (Figure 1). A total of seven sensors were used. Each sensor also had two channels for EMG data. The thigh-located sensors captured the EMG signals of the rectus femoris and the joint activity of the hamstring muscles. The lower leg-located sensors captured the EMG signals of the anterior tibial muscle and the joint activity of the external and internal heads of the triceps femoris. Disposable Medico electrodes were placed according to the SENIAM guidelines [26]. We analyzed the maximum amplitude of each muscle, μV, based on smoothed rectified EMG normalized to gait cycle, and did likewise with the goniograms.

The subjects’ upright standing positions with straightened hips and knees were assumed to be neutral (calibration position). Next, we recorded biomechanical parameters during walking. The subjects were instructed to repeatedly walk a distance of 10 m at an arbitrary pace, turning around at the end of the distance and continuing to walk. Steps with unsteady parameters (acceleration or deceleration) were automatically excluded from analysis. The remaining walking cycles were calculated. On average, recording was completed upon reaching 30 walking cycles or more. The software was based on a verified neural network algorithm for walking cycle (WC) analysis. It calculated the WC of each leg and other WC parameters. The following biomechanical parameters were selected for the subsequent analysis.

Temporal parameters: WC duration, s; rhythm (symmetry) factor (RF) (the ratio of longer to shorter stance phase). Individual WC phases and periods were measured as % of WC: stance phase (SP), single support (SS), double support (DS), and the parameter of the beginning of the contralateral leg WC (the beginning of the second double support phase, SDS).

Spatial gait parameters: largest distance between foot and floor in swing phase (foot clearance, FC), cm; walking speed (V), km/h.

Kinematic parameters were captured for the hip, knee and ankle joints in the sagittal plane (flexion–extension), the joint goniogram was plotted over a walking cycle, and the following parameters were calculated automatically.

For the hip joint: maximum amplitude over WC (HA, degrees) and the phase of maximum extension (HP).

For the knee joint: first flexion amplitude (Ka1) and its phase (Kp1), extension amplitude (Ka2) and its phase (Kp2), swing flexion amplitude (Ka3) and its phase (Kp3).

For the ankle joint: amplitude (AA) over WC.

The envelope EMG was analyzed for maximum amplitudes developed over WC, μV, by the tibialis anterior muscle (TA), calf muscles, Gastrocnemius (GA), rectus femoris muscle (RF) and hamstring muscles (HM).

### 2.2. BFB Training Sessions

The BFB training used in the study had to be within the capabilities of our patients and completed during their hospital stay. The duration of hospitalization varied depending on the hospitalization channel, and influenced the number of training sessions. The minimum number of sessions was eight. The mean number of sessions was 9.2 ± 2.12 (8–11). This was the reality in which we worked. The majority of patients received 9–10 training sessions. For this reason, we considered the variability in this parameter to be acceptable. Moreover, as analyzed earlier in the Introduction, there is no consensus on the ideal number of sessions. For a fully eligible patient (i.e., meeting all of the inclusion and none of the exclusion criteria), the duration of a training session largely depended on their condition at the time of the session. Therefore, we did not run the sessions for a specified period of time, e.g., 30 min [17], but rather until the patient’s first complaints of fatigue. Technically, we had the opportunity to observe in online mode changes in the trained parameter, including its reaching the current “ceiling” or undergoing an increase in variability with severe fatigue. However, this added uncontrolled variables to the study.

We also took into account the absence of change in training parameters, loss of walking rhythm, and increase in the range of change of the training parameter (all this information was displayed on the operator’s monitor). The mean session duration was 17:51 min (15:14 to 20:30).

**Figure 2:**
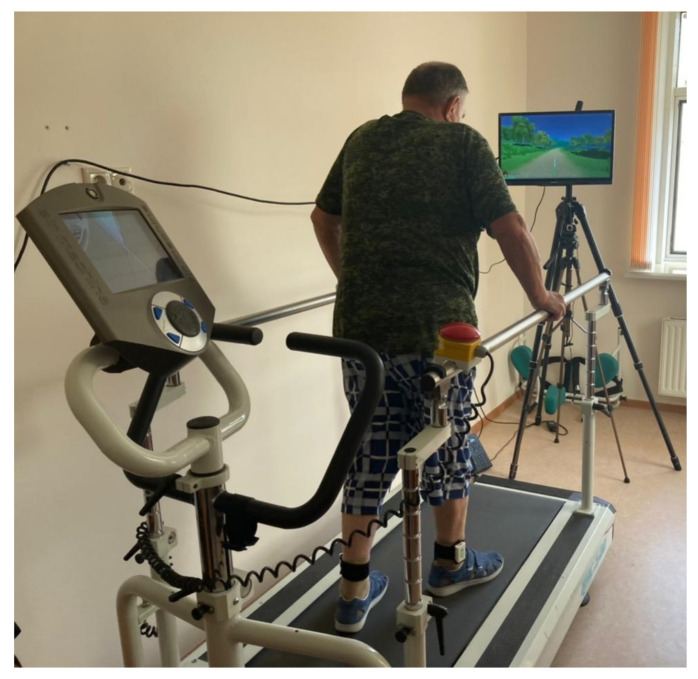
Training process. The patient is on a treadmill. Inertial sensors are installed on his shins to register gait parameters. Before him is a screen with a virtual task.

For the BFB training we used the same Stadis system, which had all necessary functionality for the training of walking based on the selected target parameter. Each training session started with a two-minute assessment of the spatiotemporal gait parameters while walking on the treadmill. The gait was assessed using two Neurosens sensors attached with elastic cuffs to the outer ankle. During the first stage, the patient—with the attached sensors—stood on the treadmill, which was then turned on and set to a speed comfortable for the patient. After that, a 2-min walking screening test was conducted. The displayed test report presented spatiotemporal gait parameters with flagged abnormal values. Next, the training parameter—stance phase in this study—was selected and a BFB training session was initiated (Figure 2). During the session, the selected parameter was displayed as columns with a marked specified range of change. An out-of-range value was shown as an error and caused a slowdown in the movement around the virtual environment and in the completion of the set task in the virtual environment. Depending on the virtual environment used, there were also other details reflecting the test performance. The automatic training algorithm was configured such that in the case of a successful completion, the range of change was adjusted towards greater symmetry of the parameters. A training session automatically continued until the patient was tired. If necessary, the range of change of the selected parameter could be adjusted manually.

The study used an Air Machine Runner RHC 500 treadmill. The training speed increased from session to session and, on average, ranged from 1.39 ± 0.44 km/h to 1.71 ± 0.42 km/h.

For convenient use in the laboratory setting, since the optimal position of the monitor for the BFB environment was at the back of the treadmill, the backward mode was used. Given the slow walking speed of the patients, the backward mode was sufficient.

The study was carried out in accordance with the ethical principles of the Declaration of Helsinki, obtaining the subjects’ written informed consent, and was approved by the Local Ethics Committee of FNKC FMBA (No. 7 of 19.07.2021).

### 2.3. Statistical Analysis

The generated data were processed with the Statistica 12 software package. Standard ANOVA methods were used to calculate the mean values and standard deviations. The significance of differences was assessed using the Wilcoxon–Mann–Whitney test with *p* < 0.05. The tested parameters were compared for paretic versus contralateral side and versus healthy control.

## 3. Results

The changes over time in patients’ conditions, as assessed by clinical scales, are presented in Table 1.

The administered therapy resulted in significant improvement as assessed by the TUG, HAI and BB methods (*p* < 0.05).

The results of the analysis of spatiotemporal gait parameters are presented in Table 2.

Walking cycle, WC, demonstrated a significant increase. Gait rhythm (characterized by rhythm factor, RF) remained less than in healthy controls (*p* < 0.05). Foot clearance, FC, on the paretic side was significantly smaller than in healthy controls or on the contralateral side before and after therapy (*p* < 0.05). Stance phase, SP, on the contralateral side was significantly greater than in healthy controls before and after therapy (*p* < 0.05). The SP of the paretic limb was also significantly smaller than on the contralateral side before and after therapy (*p* < 0.05). The single support phase, SS, of the paretic limb was significantly smaller than in healthy controls and on the contralateral side before and after therapy (*p* < 0.05). Double support phase, DS, demonstrated a significant increase versus healthy controls on both sides, before and after therapy (*p* < 0.05). The beginning of second double support (SDS) on the contralateral side before and after therapy was significantly greater compared to healthy controls (*p* < 0.05). For the paretic side before and after therapy, the same parameter was significantly smaller than in healthy controls or on the contralateral side (*p* < 0.05).

The kinematic parameters for the hip, knee and ankle joints are presented in Table 3.

Before therapy, the contralateral hip amplitude (HA) was significantly smaller, and the moment (phase) of its maximum extension (HP) occurred later than in healthy controls (*p* < 0.05). The hip amplitude of the paretic limb was significantly smaller than in healthy controls or on the contralateral side before and after therapy (*p* < 0.05). Before therapy, the maximum hip extension (HP) of the paretic limb occurred earlier than contralaterally (*p* < 0.05). After therapy, the moment (phase) of full extension (Hp) of the paretic limb occurred later than before therapy (*p* < 0.05).

Before and after therapy, the Ka1 amplitudes of both lower limbs were significantly smaller than in healthy controls (*p* < 0.05). After therapy, the Ka1 of the paretic limb increased significantly (*p* < 0.05). The moment of knee flexion (Kp1) occurred significantly earlier on the paretic side before therapy, and on the contralateral side after therapy (*p* < 0.05). The amplitude Ka2 of both lower limbs before and after therapy was significantly smaller than in healthy controls (*p* < 0.05). Before therapy, the amplitude Ka2 of the paretic limb was significantly smaller than that of the contralateral limb (*p* < 0.05). After therapy, the Ka2 of the paretic limb was significantly greater than before therapy (*p* < 0.05). After therapy, the moment of the paretic knee extension (Kp2) occurred significantly later than in healthy controls or contralaterally (*p* < 0.05). The swing amplitudes (Ka3) of both limbs before and after therapy were significantly smaller than in healthy controls (*p* < 0.05). The swing amplitude (Ka3) of the paretic limb before and after therapy was significantly smaller than on the contralateral side (*p* < 0.05). The flexion phase (Kp3) of the contralateral knee before and after therapy occurred later than in healthy controls (*p* < 0.05). The flexion phase (Kp3) of the paretic limb before and after therapy occurred earlier than on the contralateral limb (*p* < 0.05).

Before and after therapy, the ankle joint amplitudes on both sides were significantly smaller than in healthy controls (*p* < 0.05). The ankle amplitude on the paretic side was smaller than on the contralateral side (*p* < 0.05) before and after therapy.

The results of the study of the EMG activity of the muscles of interest are shown in Table 4.

Before and after therapy, the maximum electrical activity of m. Gastrocnemius on the paretic side was significantly lower than in healthy controls (*p* < 0.05) or contralaterally (*p* < 0.05). After therapy, the maximum electrical activity of hamstring muscles of the paretic limb was significantly lower than in healthy controls (*p* < 0.05), and significantly lower than on the contralateral side (*p* < 0.05).

## 4. Discussion

There was not a single negative response to BFB training among the study patients, either during the sessions or afterwards. Although patients noted fatigue by the end of the sessions, their hemodynamic parameters remained within acceptable ranges.

The outcomes of our study can be characterized as a slight improvement in walking function, as also supported by the TUG, HAI and BB assessments. Such changes are typical for the studied condition and three-week inpatient therapy.

The spatiotemporal parameters of walking showed the following regularities. Firstly, there was a whole syndrome complex of slow walking, characterized not only by a lower speed, but also by a longer WC, increased DS and, partly, increased SP. Secondly, there were asymmetries specific to stroke and, in particular, to hemiparesis: SP asymmetry with a longer duration on the unaffected side (the unaffected side thus took on a greater role in the support function). At the same time, the parameters on the paretic side were within the normal ranges for slow walking [27] both before and after the BFB training course. Statistical analysis did not detect any change in SP due to BFB training on any side.

The parameters SS and SDS also showed an asymmetry typical for the condition under study, which is the consequence of the SP asymmetry for the SS parameter and the early heel strike of the contralateral foot and late one of the paretic foot—the SDS parameter. Foot clearance (FC) was also asymmetric: it was within the normal range on the contralateral side and slightly but significantly below it on the paretic side.

The observed symptoms were typical for hemiparetic patients and did not change significantly as a result of the study therapy.

More noticeable changes were seen in the function of the joints. While before therapy the contralateral hip amplitude had been significantly lower than in healthy controls, after therapy there was no significant difference from healthy controls. The same was true for the maximum extension phase on the contralateral side (TP), which returned to the normal range. The TP on the paretic side increased to normal, which was statistically significant versus the pre-treatment value, *p* < 0.05.

The changes were even more significant for the knee joint. The first flexion amplitude (Ka1) significantly increased from the pre-treatment value *p* < 0.05. Its phase (Kp1) returned to the normal range and no longer differed significantly from healthy controls, *p* > 0.05. Importantly, before therapy, the knee extension amplitude in the middle of SP was negative (knee hyperextension, i.e., passive closure) [28]. After therapy, it remained negative (without change on the contralateral side) but decreased in absolute magnitude significantly on the paretic side *p* < 0.05. In addition, the phase of the extension (Kp2) increased significantly, i.e., the movement required more time. However, the EMG study showed no abnormal phase activity in the muscles that actuate the knee joint. This finding requires further research.

Neither the amplitude (Ka) nor the phase of the main knee flexion showed any significant changes.

The ankle joints demonstrated a decrease in amplitude, with larger values on the paretic side.

Thus, the function of the joints was characterized by kinematic changes typical for this category of patients, with insignificant but distinct improvement due to the therapy. These findings are supported by some evidence from the literature [10,29,30].

With regard to muscle function, the observed significant decrease in the function of m. Gastrocnemius and the hamstring muscles on the paretic side remained without change by the end of the treatment course. In this case, the EMG study did not confirm the detected changes in other parameters. It is rather difficult to compare this finding with other results. When the EMG activity of the triceps tibia muscle was used in a BFB system [31], the activity was not analyzed as the study variable.

## 5. Conclusions

The conducted study showed that gait training in the early recovery period after stroke can cause positive changes in walking function. Among stroke patients in the early recovery period, however, the changes in the functional parameters over the studied treatment period were minor. They were quite detectable both by traditional tests and clinical scores and by instrumental gait analysis.

The slight improvement in gait function in the study suggests that a possible increase in the number of training sessions may be beneficial. In the context of this study, there was little potential for increasing the number of sessions during the inpatient treatment stage. In future studies, the BFB training can be continued on an outpatient basis.

Technically, we could use different individual walking parameters as the basis for the BFB training. The feasibility and efficacy of their use for targeted correction need further research. Concerning the use of SP as the training parameter, there was no unambiguous response in this parameter in the study. The changes in the functional parameters over the treatment period were relatively small, and the inter-patient variability in the parameters was quite high. We suggest that the design of a continuation of this study should have tighter boundaries, both in the selection of patients and in the conducting of BFB training. We were able to observe the change in the trained parameter during the entire session. This made it possible to assess the moment when the parameter changes in the desired direction stopped. With the onset of fatigue, the variability of the parameter could significantly increase. The presence of objective feedback on the dynamics of changes in the trained parameter allowed us to assume that it is possible to focus on the patient’s individual response to training. In addition, the very individual reactions of the patient with respect to biomechanical parameters of walking during training require a separate study. For this reason, further research will need to be carried out under the most standardized conditions, including the duration and number of training sessions.

It may be useful to monitor gait parameters immediately before and after BFB training, which is technically possible. This is another possible design change for future research. That will allow for analysis of the immediate result of each session. The planning for the continuation of this study should include a reference group without BFB training. While it is feasible to slightly increase the number of BFB training sessions during the inpatient period, it is technically difficult to continue them on an outpatient basis. Not all patients can be provided with an adequate outpatient treatment stage. There are also certain restrictions on accessing hospital services during the COVID-19 pandemic.

## Figures and Tables

**Table 1:** Study outcomes, according to different clinical scales.

	TUG, s.	HAI	BB	SBT
Before	23.15 ± 6.41	3.35 ± 0.49	45.70 ± 6.43	3.60 ± 0.68
After	19.60 ± 5.11 ^&^	2.80 ± 0.70 ^&^	48.95 ± 5.34 ^&^	3.80 ± 0.52

^&^—significant, *p* < 0.05 (compared to same parameter before therapy).

**Table 2:** Spatiotemporal gait parameters.

Parameter	Before		After		Healthy Control
	Contralateral	Paretic	Contralateral	Paretic	
WC, s	1.56 ± 0.32 *	1.56 ± 0.33 *	1.53 ± 0.33 *	1.53 ± 0.32 *	1.09 ± 0.07
SP, %	72.78 ± 5.63 *	63.51 ± 4.35 ^#^	71.18 ± 4.89 *	64.46 ± 3.63 ^@^	62.85 ± 1.51
SS, %	36.13 ± 4.29	27.13 ± 6.01 *^#^	35.76 ± 3.64	29.12 ± 4.66 *^@^	37.21 ± 1.40
DS, %	36.66 ± 8.33 *	36.39 ± 8.05 *	35.42 ± 6.76 *	35.5 ± 6.70 *	25,63 ± 2,75
SDS, %	54.23 ± 4.26 *	45.49 ± 4.38 *^#^	52.87 ± 3.67 *	47.43 ± 3.61 *^@^	49.91 ± 0.41
FC, cm	12.25 ± 1.80	9.90 ± 3.11 *^#^	12.65 ± 1.60	10.20 ± 3.00 *^@^	12.55 ± 2.06
V, km/h	2.10 ± 0.77		2.32 ± 0.93		4.42 ± 0.56
RF	0.75 ± 0.17 *		0.81 ± 0.13 *		0.98 ± 0.01

*—significant, *p* < 0.05 (compared to the same parameter in the healthy control group); ^#^—significant, *p* < 0.05 (compared to the same parameter on the contralateral side before therapy); @—significant, *p* < 0.05 (compared to the same parameter on the contralateral side after therapy).

**Table 3:** Amplitude and phase parameters of hip, knee and ankle movement.

Parameter	Before		After		Healthy Control
	Contralateral	Paretic	Contralateral	Paretic	
HA	29.75 ± 3.82 *	24.30 ± 5.56 *^#^	31.15 ± 4.91	24.15 ± 5.88 *^@^	33.16 ± 4.78
HP	56.99 ± 4.14 *	51.71 ± 4.69 ^#^	55.48 ± 5.54	54.66 ± 4.55 ^&^	53.21 ± 3.04
Ka1	7.28 ± 4.32 *	5.76 ± 4.61 *	7.09 ± 5.67 *	8.11 ± 5.65 *^&^	14.60 ± 3.47
Kp1	10.87 ± 4.24	9.42 ± 3.97 *	9.33 ± 3.64 *	11.46 ± 5.28	13.10 ± 3.52
Ka2	−2.14 ± 9.13 *	−6.06 ± 6.64 *^#^	−2.73 ± 10.72 *	−1.68 ± 7.46 *^&^	4.77 ± 4.80
Kp2	37.01 ± 13.70	38.89 ± 7.03	34.44 ± 7.86	43.57 ± 8.68 *^@^	36.50 ± 4.01
Ka3	45.22 ± 9.88 *	25.43 ± 12.94 *^#^	47.27 ± 10.31 *	29.04 ± 14.61 *^@^	56.50 ± 7.33
Kp3	76.81 ± 4.38 *	67.99 ± 5.07 ^#^	76.09 ± 3.91 *	69.43 ± 5.08 ^@^	69.13 ± 2.92
AA	25.80 ± 5.61 *	22.40 ± 8.29 *^#^	26.75 ± 5.97 *	22.85 ± 6.16 *^@^	33.42 ± 6.25

*—significant, *p* < 0.05 (compared to the same parameter in the healthy control group); ^#^—significant, *p* < 0.05 (compared to the same parameter on the contralateral side before therapy); ^@^—significant, *p* < 0.05 (compared to the same parameter on the contralateral side after therapy); ^&^—significant, *p* < 0.05 (compared to the same parameter before therapy).

**Table 4:** Maximum EMG amplitudes of the studied muscles.

Muscle	Before		After		Healthy Control
	Contralateral	Paretic	Contralateral	Paretic	
TA	141.35 ± 56.38	110.40 ± 59.46	132.75 ± 48.31	111.25 ± 54.49	135.45 ± 29.03
GA	115.70 ± 60.64	54.45 ± 35.33 *^#^	106.80 ± 54.15	69.00 ± 52.80 *^@^	118.15 ± 44.48
RF	57.35 ± 23.23	50.55 ± 39.24	73.50 ± 30.64	57.05 ± 37.05	67.60 ± 43.82
HM	82.20 ± 39.80	52.05 ± 36.11 ^#^	82.55 ± 40.20	48.35 ± 33.17 *^@^	75.00 ± 27.22

*—significant, *p* < 0.05 (compared to the same parameter in the healthy control group); ^#^—significant, *p* < 0.05 (compared to the same parameter on the contralateral side before therapy); ^@^—significant, *p* < 0.05 (compared to the same parameter on the contralateral side after therapy).

## Data Availability

The data presented in this study are available on request from the corresponding author.
